# Symptomatic bilateral pulmonary embolism without deep venous thrombosis in an adolescent following arthroscopic anterior cruciate ligament reconstruction: a case report and review of the literature

**DOI:** 10.1186/s13256-018-1726-8

**Published:** 2018-07-06

**Authors:** Jonathan Bourget-Murray, Mathew A. Clarke, Sydney Gorzitza, Lisa A. Phillips

**Affiliations:** 1 Cumming School of Medicine, University of Calgary, Calgary, AB Canada; 2 College of Medicine, University of Saskatchewan, Saskatoon, SK Canada; 3 Department of Surgery, Section of Orthopedic Surgery, University of Calgary, Calgary, AB Canada; 4 Department of Surgery, Division of Pediatric Orthopedic Surgery, Alberta Children’s Hospital, 2888 Shaganappi Trail NW, Calgary, AB T3B 6A8 Canada

**Keywords:** Pulmonary embolism (PE), Deep venous thrombosis (DVT), Knee arthroscopy, Thromboprophylaxis, Anterior cruciate ligament reconstruction

## Abstract

**Background:**

Venous thromboembolism, specifically pulmonary embolism, is a rare complication following elective pediatric orthopedic surgery. Bilateral pulmonary embolism with associated pulmonary hemorrhage/infarct without concomitant deep vein thrombosis has never been reported following routine anterior cruciate ligament reconstruction in an adolescent.

**Case presentation:**

A 16-year-old white girl presented with acute onset shortness of breath and pleuritic chest pain 6 days following elective anterior cruciate ligament reconstruction. After performing a thorough work-up, she was diagnosed as having provoked bilateral pulmonary embolism with associated pulmonary hemorrhage without concomitant deep vein thrombosis. She was treated successfully with 3 months of anticoagulation therapy with daily Lovenox (enoxaparin) injections.

**Conclusions:**

Symptomatic bilateral pulmonary embolism may have a good prognosis if it is diagnosed early and treated appropriately. It is important to appreciate the risk of provoked thromboembolic events in healthy adolescents undergoing arthroscopic knee surgery.

## Background

Out-patient pediatric knee arthroscopy procedures are increasingly performed [1]. These surgeries are generally considered safe and the incidence of postoperative venous thromboembolism (VTE) is estimated to be approximately 0.25% [2]. Needless to say, few patients ever become symptomatic or suffer from pulmonary emboli (PE). To the best of our knowledge, there have been no published cases of adolescent patients with symptomatic PE after routine arthroscopic anterior cruciate ligament (ACL) reconstruction.

We present the clinical history and outcomes of a 16-year-old girl who suffered from symptomatic bilateral lower lobe PE with associated pulmonary hemorrhage/infarction following routine ACL reconstruction with bone-patellar tendon-bone autograft. The purpose of this case report is to discuss a rare thromboembolic complication following routine arthroscopic ACL reconstruction in a previously healthy adolescent. The findings demonstrate that despite being perceived as a relatively low risk surgical procedure, it is important to appreciate the risk of life-threatening thromboembolic complications associated with arthroscopic knee surgery.

## Case presentation

A 16-year-old white girl presented to our academic children’s hospital on postoperative day six with a chief complaint of shortness of breath and pleuritic chest pain. She had recently undergone an uncomplicated elective ACL reconstruction with a bone-patellar tendon-bone autograft and lateral meniscus repair with a single FAST-FIX suture (Smith & Nephew, Inc., Andover, MA, USA). The surgery was performed under a general anesthetic as well as a femoral canal block as per our usual protocol. There were no intraoperative complications. Total tourniquet time was 127 minutes at 250 mmHg. Her postoperative course was uncomplicated. She was discharged later that day as her pain was well controlled and she was cleared by physiotherapy. She was encouraged to be weight-bearing as tolerated while limiting her range-of-motion to 0° to 90° of knee flexion for 6 weeks, given her meniscal repair.

On the day of surgery, she weighed 65.3 kg and her height was 165.7 cm. Her past medical history was significant for acne which was controlled with tetracycline. She was on no other regular medication nor did she take a birth control pill. She was a non-tobacco smoker. She had no family history of anesthetic or hematological issues.

**Fig. 1:**
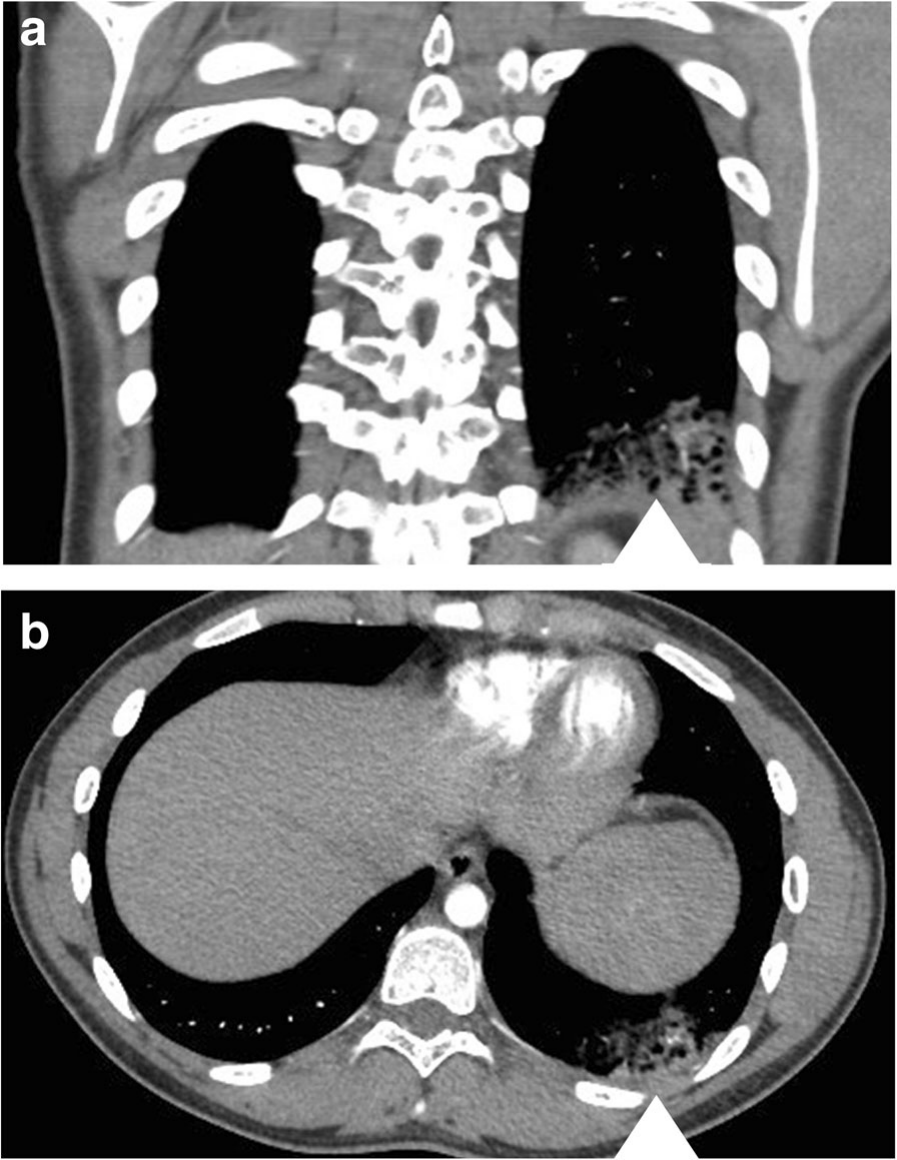
Bilateral lower lobe pulmonary embolism more numerous on the left. There is an associated opacity in the left lung base probably representing pulmonary hemorrhage/infarction. **a** Coronal and **b** axial views showing multiple filling defects in the left lower lobe pulmonary arteries as well as a groundless opacity (*white arrowhead*) compatible with pulmonary hemorrhage related to the pulmonary embolism. Hematology decided not to rescan with computed tomography to show resolution of the pulmonary embolism following 3 months of anticoagulation therapy

She presented to our Emergency Department on postoperative day six with progressively worsening pleuritic chest pain and shortness of breath. Her vital signs upon presentation were: temperature, 36.8 °C; pulse, 104 beats per minute (bpm); respiration rate, 20/minute; blood pressure, 135/76 mmHg; and O_2_ saturation of 96% on room air. Baseline laboratory work was drawn (Table 1) and a computed tomography (CT)-PE study was performed. This revealed bilateral lower lobe emboli with moderate clot burden and an associated opacity in the left lung base in keeping with pulmonary hemorrhage/infarction (Fig. 1). She was subsequently admitted and managed medically with a weight-based dose of Lovenox (enoxaparin) twice daily in consultation with Hematology. She was discharged home 3 days later and would successfully complete 3 months of anticoagulation therapy without any further complications. Anticoagulation treatment was stopped after 3 months as per Hematology.

**Table 1:** Baseline blood work drawn upon initial emergency visit

Tests	Results	Normal range
Hemoglobin	133	120–160 (g/L)
Hematocrit	0.38	0.36–0.48 (L/L)
RBC	4.7	4.0–5.6 (10^12^/L)
MCV	83	82–100 (fL)
MCHC	345	320–360 (g/L)
RDW	12.5	11.0–16.0 (%)
Platelet Count	259	150–400 (10^9^/L)
WBC	*11.5*	4.0–11.0 (10^9^/L)
Creatinine	62	40–100 umol/L
INR	1.0	0.9–1.1
PTT	30.6	27.0–37.0
Troponin	6	0–14 (ng/L)
Fibrinogen	*5.4*	1.6–4.1 (g/L)
Anti-factor Xa	0.69	Anti-Xa U/mL
D-dimer	*2.71*	0.00–0.45 (mg/L FEU)

*INR* international normalized ratio, *MCHC* mean corpuscular hemoglobin concentration, *MCV* mean corpuscular volume, *PTT* partial thromboplastin time, *RBC* red blood cells, *RDW* random distribution of red cell width, *WBC* white blood cells

Italicized results represent significant laboratory findings

At her 12-month follow-up, she was asymptomatic and found to have no concerning cardiorespiratory issues. Both an echocardiogram and a pulmonary function test were performed, and found to be within normal limits. Hematology decided not to rescan her in order to assess resolution of the PE given her clinical profile. They have investigated for some treatable causes of spontaneous or acquired thromboembolism (Table 2). Results for lupus anticoagulant and anti-phospholipid syndrome as well as for paroxysmal nocturnal hemoglobinuria all returned negative. Admittedly, no further testing was performed to identify causes of inherited thrombophilia (that is, factor V Leiden, prothrombin G20210A, protein C, protein S, and/or antithrombin levels), following a thorough conversation with the family, as this would not have changed treatment pathway irrespective of results.

**Table 2:** Additional blood work drawn by hematology to rule out any treatable causes of spontaneous or acquired thromboembolism

Test	Results
Anti-beta 2 glycoprotein 1	Negative
Anti-nuclear antibody	Positive
ANA pattern 1	Speckled
ANA titer	1:40
Cardiolipin antibodies	Negative
PNH panel	Not detected
Anti-factor Xa	0.83
Lupus-type inhibitor TTI	Not detected
Lupus-type inhibitor RVV	Not detected
D-dimer	0.13

Results for lupus anticoagulant and anti-phospholipid syndrome as well as for paroxysmal nocturnal hemoglobinuria.

*ANA* anti-nuclear antibody, *PNH* paroxysmal nocturnal hemoglobinuria

The hematological team did recommend to our patient that she did not use an estrogen-containing oral contraceptive pill, favoring a progesterone-only oral contraction or a progesterone-containing intrauterine device. From an orthopedic standpoint, she is ambulating independently and cleared for return to sport without any restrictions.

## Discussion

The purpose of this case report is to present a case of a previously healthy adolescent who suffered from symptomatic bilateral PE without evidence of lower extremity deep venous thrombosis (DVT) following routine ACL reconstruction. Despite being perceived as a relatively low risk surgical procedure, it is important to appreciate the risk of life-threatening complications associated with surgery. As reported here, a symptomatic PE can occur regardless of age and perioperative risk profile.

Sabharwal *et al.* reported on a follow-up web-based questionnaire from members of the Pediatric Orthopedic Society of North America looking at VTE following nonspecific surgery in pediatrics [3]. Results extrapolated from 46 children revealed that the most commonly cited predisposing factors for the development of VTE were having undergone lower extremity surgery and being an adolescent. More importantly, 26% of the patients in their case series were diagnosed with PE without evidence of DVT [3]. Despite PE being extremely uncommon among children, it is important to properly diagnose and treat these patients as the mortality rate following PE in children has been reported to approach 16% [4].

Owing to the low operative risk associated with knee arthroscopy, use of pharmacologic VTE prophylaxis in the postoperative period after ACL reconstruction has been thought to be unnecessary, so much so that many surgeons have discontinued their routine use. At our tertiary pediatric university-affiliated hospital, routine postoperative thromboprophylaxis is not routine practice. The most recent clinical practice guidelines from the American College of Chest Physicians for VTE treatment recommend against the routine use of pharmacologic VTE prophylaxis after knee arthroscopy in patients without a prior personal history of VTE [5]. Upon thorough review of the PubMed and MEDLINE databases, it is evident there is a lack of evidence evaluating the risks and benefits associated with VTE prophylaxis in the adolescent population following knee arthroscopy.

It is common practice to have all our patients who are undergoing an ACL reconstruction receive both a regional and general anesthetic. The former aims to optimize the patient’s postoperative pain profile. Admittedly, it is unclear if regional blocks influence the development of VTE following ACL reconstruction. Although not specific to pediatric patients, Hetsroni *et al.* reviewed 418,323 patients who underwent knee arthroscopy and identified those admitted within 90 days of surgery due to PE. Their multiple logistic regression model showed that type of anesthesia was not remotely associated with development of PE [6].

Given that our patient suffered from what was considered a provoked PE, the hematological team at our academic center do not routinely screen for common hereditary hypercoagulability disorders because this will often not change medical management. However, as with our patient and her family in the present case, a comprehensive meeting will be held in which the risks and benefits of further investigations are discussed. Common causes of inherited thrombophilia, such as heterozygous factor V Leiden mutation, are explained. However, it is clearly explained that regardless of knowing the mutational status of the patient, this often does not change the treatment pathway. Knowing so, however, could be beneficial to patients, in order to modify their day-to-day life, as they would be at increased risk of VTE recurrence compared to those without the mutation. In this case, it was decided to not go forward with additional testing. Should this patient experience a VTE recurrence in the future, further testing would most certainly be performed.

## Conclusions

In conclusion, until further studies that examine complications associated with VTE prophylaxis become available, we do not recommend routine VTE prophylaxis for all patients having arthroscopic ACL reconstruction. We currently recommend to our patients early postoperative mobilization and consider pharmacologic VTE prophylaxis in patients who present with risk factors for developing a VTE or a personal or family history of DVT or PE.
